# Racial and Socioeconomic Disparities in California Ambulance Patient Offload Times

**DOI:** 10.1001/jamanetworkopen.2025.10325

**Published:** 2025-05-15

**Authors:** Vadim M. Shteyler, Madeline Feldmeier, Richard Julian G. Bagay, Dustin Ballard, Christopher Colwell, Renee Y. Hsia

**Affiliations:** 1 Division of Pulmonary, Critical Care, Allergy, and Sleep Medicine, University of California, San Francisco; 2 Philip R. Lee Institute for Health Policy Studies, University of California, San Francisco; 3 Department of Emergency Medicine, University of California, San Francisco; 4 School of Public Health, University of California, Berkeley; 5 Department of Emergency Medicine, Kaiser Permanente, San Rafael, California

## Abstract

**Importance:**

Patient demographic and socioeconomic characteristics are associated with differential delays in access to emergency care. However, less is known about the association between demographic, socioeconomic, and emergency medical services (EMS) agency factors and ambulance patient offload times (APOT), a critical measure of timely access to emergency care.

**Objective:**

To determine the association between ambulance offload times and demographic, socioeconomic, and EMS agency factors, uncover disparities in offload delays, and investigate factors associated with long APOT.

**Design, Setting, and Participants:**

This retrospective cohort study was conducted using offload data from the California EMS Authority and local EMS agencies (LEMSA), population data from the US Census Bureau, and demographic and socioeconomic data from the Centers for Disease Control and Prevention’s Social Vulnerability Index. California local EMS agencies participated from January 1, 2021, to June 30, 2023.

**Exposure:**

A total of 30 community demographic, socioeconomic, and LEMSA factors were assessed.

**Main Outcomes and Measures:**

The main outcome was weighted mean APOT, calculated from APOT measures obtained from the California Emergency Medical Services Authroity and LEMSAs. The association between APOT and area demographic and socioeconomic characteristics was assessed using linear regression.

**Results:**

In this retrospective study from January 1, 2021, to June 30, 2023, 5 913 399 offloads were observed across 34 California LEMSAs with median (IQR) of 0.3% (0.2%-0.4%) American Indian or Alaska Native residents, 7.1% (4.7%-1.5%) Asian residents, 2.2% (1.6%-4.9%) Black residents, 33.1% (22.5%-48.7%) Hispanic or Latino residents, 0.3% (0.2%-0.4%) Native Hawaiian or Pacific Islander residents, 4.1% (3.1%-4.9%) multiracial, and 0.4% (0.4%-0.5%) residents in the additional race category; 17.2% (14.7%-22.8%) residents living below 150% of the poverty line; and 15.7% (13.5%-20.1%) aged 65 years or older. The weighted mean (SD) APOT across California was 42.8 (27.3) minutes; the median (IQR) across LEMSAs was 27.0 (15.5-48.3) minutes. Sequential unadjusted linear regressions of the 30 characteristics revealed 11 that were significantly associated with APOT. Least absolute shrinkage and selection operator penalization identified Black race, being aged 65 years or older, and total offloads (all log-transformed) as the variables most associated with APOT. A 3.3% absolute increase in Black residents, from 1.6% (25th percentile) to 4.9% (75th percentile), was associated with an unadjusted 17.4-minute (95% CI 10.3-24.5) increase in APOT. This increase remained significant, at 11.75 (95% CI 1.9-21.6) minutes, after adjustments for demographic, socioeconomic, and LEMSA characteristics.

**Conclusions and Relevance:**

This cohort study of 5.9 million ambulance offloads found that the proportion of Black residents was the factor most significantly associated with longer APOT. These findings have direct implications for patients, EMS systems, and hospitals, as APOT may be an important component of health disparities in emergency care.

## Key Points

**Question:**

Are ambulance patient offload times associated with community demographic, socioeconomic, and emergency medical services (EMS) agency factors?

**Findings:**

In this cohort study of 5.9 million ambulance offloads across 34 California EMS agencies, Black race was found to be the factor most associated with increased offload times; a 3.3% increase from the 25th to 75th percentile of the proportion of Black residents was associated with a 17.4-minute unadjusted and 11.8-minute fully adjusted increase in ambulance patient offload time.

**Meaning:**

These findings suggest ambulance offload delays may be an important factor when considering health disparities in emergency medicine.

## Teaser

This cohort study evaluates the demographic, socioeconomic, and emergency medical services agency factors associated with ambulance patient offload times in California.

## Introduction

Rapid prehospital response and medical intervention are essential components of emergency care. Traditional measures of timely access to prehospital care include emergency medical services (EMS) response times, time on scene, and transport time to the hospital. A novel, distinct measure of timeliness that has recently been used to capture a more comprehensive view of emergency care access is ambulance patient offload time (APOT)—the time interval between an ambulance and patient arriving at an emergency department (ED) and ED personnel assuming the patient’s care. While response times are used to assess timely access to critical, life-saving interventions, time on scene and transport times have often been used to assess access to emergency care beyond immediate life-saving procedures provided in the prehospital environment. Prolonged APOT reflects ED and hospital system-wide strains that other measures may fail to capture. Longer APOT not only means that patients who access emergency care through the 911 system are waiting longer to receive that care, but it is also an indicator of emergency department crowding, which, in turn, has been associated with reduced quality of care and increased morbidity and mortality.^1^ Despite efforts to improve crowding in the emergency department and reduce APOT in recent years, delays have persisted and even worsened.^2^

Current literature has shown that community demographic and socioeconomic characteristics are associated with disparate delays in access to care. Lower community socioeconomic status (SES) is associated with longer EMS response and transport times for suspected stroke patients, and EMS response times to cardiac arrest calls are longer in poorer neighborhoods compared with more affluent ones.^3,4^ Furthermore, a community’s racial composition is associated with ED crowding severity, with predominantly White communities experiencing less crowding and ambulance diversion than more racially minoritized communities.^5^ Meanwhile, delays in care are consequential for all patients seeking emergency care. Longer ED wait times have been linked with a higher likelihood of poor patient outcomes and have been shown to disproportionately impact uninsured and Medicaid patients.^6^ Although research specifically on APOT is limited, studies show that longer APOT is associated with worse patient outcomes,^7^ increased response times,^8^ financial losses for EMS agencies,^9^ and reduced availability of critical care services in the surrounding community.^10^

The association between community demographic and socioeconomic factors and APOT, however, has not yet been investigated. This relationship is important because APOT is a more real-time measure of accessibility, reflecting both a patient’s individual and actual access to emergency care as well as a health system’s ability (or inability) to respond to public health demands with its services. As EDs serving racially minoritized and lower SES communities are more likely to experience crowding, capacity, strain, and surrounding hospital closures,^11^ they may also face longer APOT, which may perpetuate a spiral of delays in care and result in worse outcomes. Thus, to fulfill the public health duty of addressing sociodemographic disparities in access to care, this study examines the associations and interplay of APOT and 30 different community demographic, socioeconomic, and local EMS agency (LEMSA) factors, deepening our understanding of factors associated with differential access to timely emergency care.

## Methods

### Data Sources

This study was deemed exempt from institutional review board approval and the need for informed consent at UCSF because it did not involve human participants. The study followed the Strengthening the Reporting of Observational Studies in Epidemiology (STROBE) reporting guidelines.

We obtained APOT data from the California EMS Authority and EMS agencies. Our primary APOT data source was the EMS Authority website^12^; however, reports were unavailable for January through March 2023 and thus were supplemented by data directly from EMS agencies (further details in the eMethods in Supplement 1). We previously calculated the mean APOT, weighted for the number of observed offloads, and annual mean offload volumes per 1000 population aggregated at the LEMSA level from January 1, 2021, through June 30, 2023.^2^ Each LEMSA serves 1 or more counties in California, thus we extracted county-level population data from the US Census Bureau.^13^ We also obtained 28 county-level component measures from the 2022 Social Vulnerability Index (SVI)^14^ which represent socioeconomic, self-reported demographic and race, household, and housing type and transportation measures from the Centers for Disease Control and Prevention (CDC) website (eTable 1 in Supplement 1). County-level data (eg, number of people self-reporting Black race, county population, county area, and so forth) were summed for multicounty LEMSAs. The SVI measures were divided by LEMSA population to obtain a population proportion (reported as percentages). Measures of multiraciality (“two or more races, not Hispanic or Latino” in CDC data) and aggregated minoritized race (the sum of all reported non-White race and ethnicity categories in CDC data: “Hispanic or Latino, of any race; Black and African American, not Hispanic or Latino; American Indian and Alaska Native, not Hispanic or Latino; Asian, not Hispanic or Latino; Native Hawaiian and Other Pacific Islander, not Hispanic or Latino; two or more races, not Hispanic or Latino; other races, not Hispanic or Latino”) were excluded from adjusted analyses to reduce collinearity. The Table summarizes the overall LEMSA population characteristics and their differences across quartiles of mean APOT in California LEMSAs.

**Table.** Summary of California Local Emergency Medical Services Agency (LEMSA) Characteristics and Their Populations by Ambulance Patient Offload Time (APOT) Quartiles

Characteristic	APOT quartiles, median (IQR), min				
	1 (n = 9)	2 (n = 8)	3 (n = 8)	4 (n = 9)	Overall (n = 34)
LEMSA characteristic					
Offloads, No.^a^	35 617 (12 907-46 695)	69 963.5 (32 223-107 571)	173 634 (78 664-238 107)	310 263 (19 7765-395 543)	91 199 (36 059-243 710)
Offloads per 1000 population, No.	62 (56-75)	62 (58.3-73.3)	73 (55.3-84.3)	71 (66-76)	69.5 (56.3-75.8)
LEMSA area, square miles	2221 (752-3301)	2561 (1486.3-4270)	1444 (1167-2404)	4060 (965-8135)	1889 (858-4202)
LEMSA population, No.^a^	260 485 (137 384-445 213)	444 302 (249 357-607 849)	815 240.5 (484 620-2 231 430)	1 663 823 (116 264-2 212 611)	605 551 (262 507-1475 070)
Race and ethnicity^b^					
American Indian or Alaska Native	0.002 (0.002-0.003)	0.002 (0.002-0.003)	0.003 (0.003-0.004)	0.003 (0.002-0.005)	0.003 (0.002-0.004)
Asian	0.034 (0.029-0.062)	0.053 (0.045-0.075)	0.142 (0.068-0.248)	0.145 (0.073-0.170)	0.071 (0.047-0.151)
Black^a^	0.017 (0.015-0.018)	0.016 (0.012-0.021)	0.027 (0.021-0.046)	0.074 (0.049-0.084)	0.022 (0.016-0.049)
Hispanic or Latino	0.234 (0.166-0.351)	0.311 (0.254-0.477)	0.342 (0.225-0.440)	0.487 (0.264-0.546)	0.331 (0.225-0.487)
Minoritized race^a^^,^^c^	0.331 (0.308-0.574)	0.504 (0.378-0.662)	0.616 (0.602-0.710)	0.685 (0.594-0.723)	0.604 (0.402-0.703)
Multiracial	0.043 (0.033-0.048)	0.041 (0.033-0.047)	0.041 (0.036-0.046)	0.032 (0.030-0.053)	0.041 (0.031-0.049)
Native Hawaiian or Pacific Islander	0.003 (0.002-0.010)	0.003 (0.002-0.004)	0.003 (0.002-0.003)	0.003 (0.003-0.004)	0.003 (0.002-0.004)
Additional race categories	0.005 (0.004-0.006)	0.004 (0.003-0.005)	0.004 (0.003-0.004)	0.004 (0.004-0.005)	0.004 (0.004-0.005)
Socioeconomic status^b^					
Below 150% poverty	0.169 (0.138-0.215)	0.156 (0.151-0.180)	0.179 (0.160-0.219)	0.224 (0.194-0.238)	0.172 (0.147-0.228)
Unemployed	0.030 (0.027-0.033)	0.028 (0.024-0.030)	0.033 (0.028-0.035)	0.032 (0.031-0.036)	0.031 (0.027-0.034)
Burdened by housing cost	0.097 (0.083-0.116)	0.091 (0.089-0.099)	0.091 (0.089-0.101)	0.100 (0.093-0.105)	0.095 (0.087-0.104)
No health insurance	0.060 (0.057-0.062)	0.058 (0.047-0.069)	0.062 (0.051-0.069)	0.073 (0.046-0.080)	0.061 (0.047-0.072)
No high school diploma	0.075 (0.058-0.107)	0.079 (0.072-0.118)	0.090 (0.077-0.125)	0.111 (0.077-0.137)	0.080 (0.073-0.116)
Housing characteristics^b^					
Housing units^d^	0.429 (0.377-0.448)	0.378 (0.344-0.424)	0.358 (0.330-0.400)	0.362 (0.342-0.371)	0.368 (0.343-0.425)
Households^d^	0.381 (0.350-0.391)	0.351 (0.320-0.374)	0.338 (0.315-0.360)	0.338 (0.306-0.351)	0.350 (0.311-0.378)
Multiunit homes	0.026 (0.019-0.051)	0.034 (0.028-0.038)	0.049 (0.022-0.083)	0.048 (0.027-0.060)	0.036 (0.024-0.054)
Group homes	0.032 (0.026-0.049)	0.022 (0.016-0.042)	0.022 (0.019-0.028)	0.020 (0.016-0.025)	0.023 (0.016-0.033)
Mobile homes^d^	0.028 (0.011-0.046)	0.020 (0.014-0.026)	0.012 (0.010-0.016)	0.014 (0.006-0.020)	0.015 (0.010-0.026)
Crowded living^d^	0.017 (0.014-0.023)	0.020 (0.016-0.024)	0.026 (0.024-0.027)	0.026 (0.022-0.029)	0.024 (0.018-0.026)
Household characteristics^b^					
Age ≥65 y^a^	0.202 (0.169-0.211)	0.171 (0.158-0.201)	0.144 (0.132-0.157)	0.142 (0.122-0.146)	0.157 (0.135-0.201)
Age ≤17 y^d^	0.199 (0.196-0.211)	0.221 (0.196-0.232)	0.213 (0.200-0.267)	0.231 (0.211-0.258)	0.213 (0.198-0.252)
Single parent household^a^	0.018 (0.017-0.020)	0.017 (0.016-0.021)	0.018 (0.014-0.023)	0.020 (0.019-0.024)	0.019 (0.016-0.022)
No car/vehicle	0.020 (0.018-0.022)	0.016 (0.015-0.017)	0.018 (0.016-0.020)	0.020 (0.018-0.027)	0.019 (0.016-0.021)
No home internet	0.027 (0.021-0.048)	0.028 (0.026-0.032)	0.031 (0.022-0.033)	0.029 (0.025-0.033)	0.029 (0.024-0.033)
Civilian with a disability	0.120 (0.107-0.167)	0.122 (0.115-0.125)	0.114 (0.098-0.126)	0.112 (0.105-0.115)	0.116 (0.106-0.126)
Limited English proficiency	0.041 (0.025-0.071)	0.055 (0.042-0.102)	0.084 (0.076-0.091)	0.070 (0.060-0.092)	0.070 (0.047-0.086)
Weighted APOT, min	13.3 (10.3-13.5)	22.1 (19.8-23.5)	38 (33.6-42.8)	51.4 (50.1-54.4)	27 (15.5-48.3)

^a^ Unadjusted linear associations with statistical significance level at *P* < .01.

^b^ Race and ethnicity, socioeconomic status, housing characteristics, and household characteristics reported as proportions of total 2020 LEMSA populations.

^c^ Minoritized Race includes Hispanic or Latino (of any race); Black and African American; American Indian and Alaska Native; Asian; Native Hawaiian and Other Pacific Islander; 2 or more races; and additional races as per the US Centers for Disease Control and Prevention Social Vulnerability Index.

^d^ Unadjusted linear associations with statistical significance level at *P* < .05.

### Statistical Analysis

This exploratory analysis used linear regression to estimate the unadjusted linear trends between the weighted-mean APOT in minutes and, sequentially, each of the 28 LEMSA and 2 offload characteristics. Select variables were log or square root transformed to address linearity, homoskedasticity, and residual normality assumptions. Least absolute shrinkage and selection operator (LASSO) penalization with a lambda within 1 SE of the minimum were used to simultaneously select between different variable transformations and select the variables most associated with APOT. The R package selectiveInference estimated unbiased LASSO coefficients and 95% CIs from the resultant model. Adjusted models reflected our understanding of the confounding, interactions, or mediation effects between the significant predictors identified through LASSO penalization, other sociodemographic or LEMSA variables, and APOT. We used the 4-step mediation analysis with the mediation package in R to estimate direct and indirect effects.^15,16^ We additionally estimated the association between the Black race proportion and APOT for nested regression models, any of which may be viewed as appropriately-adjusted, but making slightly different assumptions about what plausibly confounds the association between APOT and race. The fully-adjusted model was adjusted for the (log transformed when appropriate) remaining race and ethnicity categories, the available socioeconomic variables plausibly associated with APOT, the interaction between the proportion of Black and African American, not Hispanic or Latino residents (subsequently referred to as Black) and the proportion living below 150% of the federal poverty line (the SVI measure of economic disadvantage, subsequently referred to as 150% poverty), age categories, and LEMSA characteristics. We reported results from models on a logarithmic scale as the difference in APOT for populations at the observed 25th and 75th percentiles of a covariate (eg, percentage of Black residents), with adjustment variables at their mean values. Otherwise, coefficients on the multiplicative scale are identified as such or, for ancillary analyses, significance is simply reported as *P* values. Select ancillary analyses used a calculated White race population proportion per CDC documentation.^13^

All analyses were performed with R software version 4.3.2 for Windows (R Project for Statistical Computing), with the statistical significance level set at *P* < .05. All tests were 2-tailed.

## Results

Over the study observation period from January 1, 2021, through June 30, 2023, we observed 5 913 399 offloads across 34 LEMSAs in California, with a median (IQR) of 91 199 (36 059-243 710) offloads per LEMSA (Table). The APOT weighted mean (SD) across California was 42.8 (27.3) minutes; the median (IQR) across LEMSAs was 27.0 (15.5-48.3) minutes. The Table summarizes the overall LEMSA population characteristics and their differences across quartiles of mean APOT in California LEMSAs. The median (IQR) LEMSA had 7.1% (4.7%-1.5%) Asian residents, 2.2% (1.6%-4.9%) Black residents, and 33.1% (22.5%-48.7%) Hispanic or Latino residents. Cumulatively, the LEMSAs had a median of 60.4% (40.2-70.3) of residents identifying as a minoritized race. LEMSAs with offload times in the top 2 quartiles had higher median population proportions of each of these race categories than LEMSAs in the bottom 2. The proportions of the remaining race categories did not meaningfully differ between quartiles of APOT. The median (IQR) LEMSA had 17.2% (14.7%-22.8%) of residents living below 150% the poverty line, 1.9% (1.6%-2.2%) single-parent households, 6.1% (4.7%-7.2%) without health insurance, 2.9% (2.4%-3.3%) without internet, 1.9% (1.6%-2.1%) without a vehicle, and 11.6% (10.6%-12.6%) with a disability. LEMSAs in the top 2 quartiles of APOT had higher median measures of economic disadvantage, unemployment, health uninsurance, household size, crowded living, and limited English proficiency than LEMSAs in the bottom 2 quartiles. Conversely, LEMSAs in the top 2 quartiles of APOT had lower median measures of group homes, mobile homes, high school completion, and disability prevalence. The median (IQR) LEMSA had 15.7% (13.5%-20.1%) of residents aged 65 years or older, with the LEMSAs in the highest 2 APOT quartiles having a smaller median proportion of residents aged 65 years or older than LEMSAs in the lowest 2 APOT quartiles.

Sequential unadjusted linear regression of each of the untransformed 28 LEMSA characteristics and 2 offload measures revealed 11 variables with statistically significant linear associations with APOT (designated in the Table; shown in eTable 2 in Supplement 1). Seven had positive associations: total offloads, LEMSA population, Black race proportion, minoritized race proportion, single-parent households per capita, crowded homes per capita, and the proportion of residents aged 17 years or younger. The log transformed proportion of residents living below 150% poverty was also positively associated with APOT. Four variables had negative associations: housing units per capita, households per capita, mobile homes per capita, and the proportion of residents aged 65 years or older.

LASSO penalization identified Black race (regression coefficient, 6.7; 95% CI, 0.56 to 12.1; *P* = .04), being aged 65 years or older (regression coefficient, 5.2; 95% CI, 1.9 to 8.4; *P* = .01), and total offloads (regression coefficient, −24.0; 95% CI, −38.6 to −8.4; *P* = .01) as the variables most predictive of APOT (all log-transformed and on the multiplicative scale) (eTable 3 in Supplement 1). These 3 variables, along with 150% poverty, were log transformed in subsequent adjusted models unless otherwise specified.

We estimated the change in APOT that would be associated with an increase in Black residents from 1.6% to 4.9% of the population, corresponding with an increase from the 25th to the 75th percentile of Black race proportions in the observed LEMSAs. An unadjusted model of the association between the proportion of Black residents and APOT estimated a 17.4-minute (95% CI, 10.3 to 24.5 minutes; *P* < .001) increase in APOT for this 3.3% absolute increase in Black residents.

The proportion of residents living below 150% of the poverty line did not mediate the association between Black race proportion and APOT (proportion mediated 0.05; 95% CI, −0.04 to 0.19; *P* = .31) (eTable 3 in Supplement 1). There was a statistically significant positive interaction between the proportion of Black residents and those living below 150% of the poverty line (interaction coefficient 29.05 on the multiplicative scale; 95% CI, 7.8 to 50.3; *P* = .01). The adjusted estimated APOT differs along the observed range of the proportion of Black residents when the proportion living below 150% of the poverty line is set at its observed 75th percentile or observed 25th percentile value. Notably, measures of limited English proficiency, unemployment, high school completion, health uninsurance, vehicle ownership, home internet, and disability were not associated with APOT in models including Black race proportion.

In contrast, the LEMSA proportion of residents aged 65 years or older was associated with a 17.7-minute (95% CI, 1.6 to 19.7; *P* < .001) decrease in APOT for a 25th to 75th percentile increase in the proportion of residents aged 65 years or older. Being aged 65 years or older and APOT remained significantly associated with APOT despite adjustment for uninsurance and 150% poverty, and the association between being aged 65 years or older and APOT was neither mediated by uninsurance nor 150% poverty. This suggests that a higher proportion of residents aged 65 years or older was not associated with decreased APOT through reductions in either uninsurance or economic disadvantage.

Being aged 65 years or older and APOT were no longer significantly associated after adjustment for the proportion of residents living below 150% of the poverty line and Black race or their interaction (eTable 3 in Supplement 1). Both poverty and Black race, alone and together, resulted in decreased proportions of residents aged 65 years or older. Likely, the association between being aged 65 years or older and lower APOT reflects the lower proportions of Black and economically disadvantaged residents in LEMSAs with higher proportions of residents aged 65 years or older. The proportion of residents living below 150% of the poverty line and its interaction with Black race no longer predicted the proportion of residents aged 65 years or older after further adjustment for the proportion aged 17 years or younger. The proportion of residents of Black race and its interaction with those living at 150% of the poverty line were positively associated with the proportion aged 17 years or younger. In sum, the proportion of residents aged 65 years or older likely predicted lower APOT because LEMSAs with more minors had smaller proportions aged 65 years or older and higher proportions of Black and economically disadvantaged residents.

A crude (untransformed) model showed a 0.42-minute increase in APOT for every additional 10 000 offloads (95% CI, 0.2 to 0.6; *P* < .001) in a LEMSA and a 0.42-minute increase in APOT for every additional 100 000 residents (95% CI, 0.1 to 0.7; *P* = .002) in a LEMSA (eTable 2 in Supplement 1). The association between untransformed LEMSA area or mean annual offloads per capita and APOT was not statistically significant. Counterintuitively, the (log) number of offloads was inversely associated with the fraction aged 65 years or older (coefficient −1.8 on the multiplicative scale; 95% CI −3.5 to −0.09; *P* = .047), but this association was no longer significant after adjustment for Black race proportion (coefficient −0.2 on the multiplicative scale; 95% CI, −1.8 to 1.44; *P* = .81) or White race proportion (coefficient −0.3 on the multiplicative scale; 95% CI, −4.3 to 3.6; *P* = .87).

**Figure 1.**
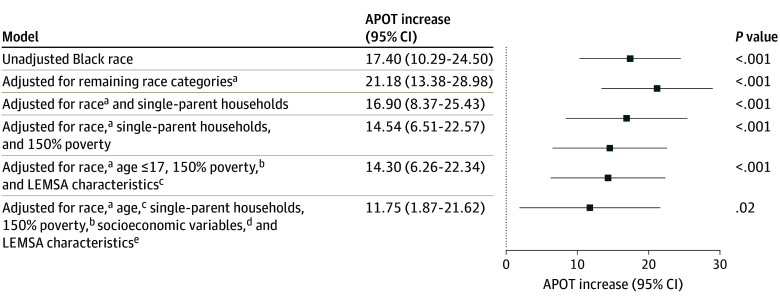
Estimated Ambulance Patient Offload Time (APOT) Change (Minutes) Associated With a Black Race Population Fraction Increase From the 25th to 75th Percentiles Observed in CA Local Emergency Medical Services Agencies (LEMSA) Figure 1 has been adjusted for suspected confounders at marginal mean levels: remaining race categories, single-parent households, 150% poverty, age categories, LEMSA characteristics, and socioeconomic variables. ^a^Remaining race categories: population fraction that is Hispanic or Latino, Asian (not Hispanic or Latino), American Indian or Alaska Native (not Hispanic or Latino), Native Hawaiian or Pacific Islander (not Hispanic or Latino), or additional races (not Hispanic or Latino). ^b^Adjusted for both 150% poverty and its interaction with Black race proportion. ^c^Age categories: population fraction that is aged 65 years and older or 17 years and younger. ^d^Socioeconomic variables: population proportion that is unemployed, burdened by housing cost, has limited English proficiency, has no health insurance, has no High School diploma, and has no vehicle. ^e^LEMSA characteristics: population, number of offloads, and area (square miles).

**Figure 2.**
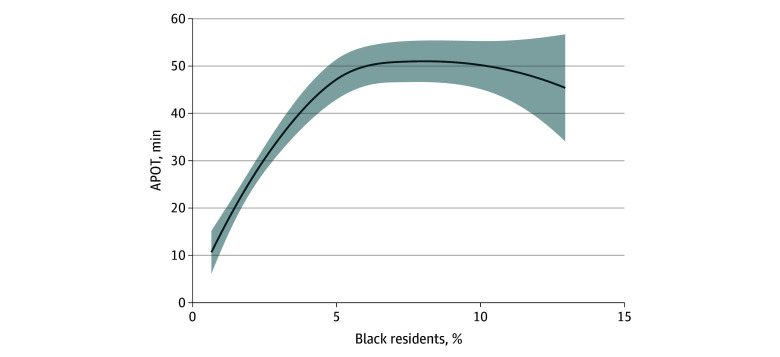
Adjusted Association Between Black Resident Proportion and Ambulance Patient Offload Time (APOT) The association between APOT (minutes) and percentage Black race, estimated from the model fully adjusted for remaining race and age categories, single parent households, all available socioeconomic and LEMSA characteristics, and the interaction between Black race and economic disadvantage.

**Figure 3.**
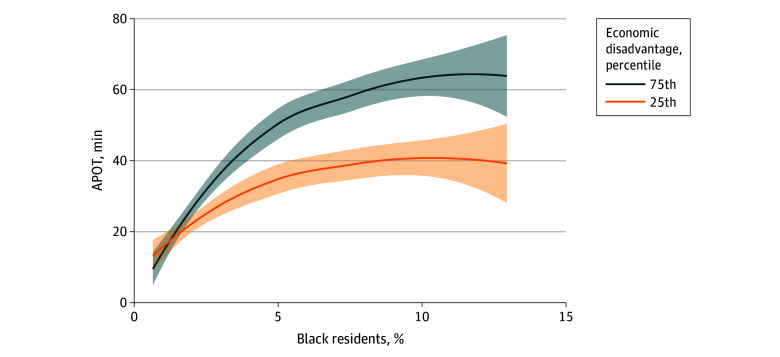
Estimated Association of Black Race Proportion and Ambulance Patient Offload Time (APOT), Adjusted for Local Emergency Medical Services Agency (LEMSA) Population, Offloads, and Area, at the 25th and 75th Percentiles of Residents Living Below 150% Poverty Estimated associations between the proportion of residents reporting Black race and APOT for the 75th percentile of economic disadvantage (blue) and then 25th percentile of economic disadvantage (orange) of observed LEMSAs, adjusted for LEMSA population, offloads, and area.

Figure 1 shows the predicted APOT change over the 3.3% absolute increase in Black residents after nested adjustment for suspected confounders set at their mean levels. After full adjustment for confounders by race and age categories, single-parent households, all available socioeconomic variables, and LEMSA characteristics, and for the interaction between Black race and those living at 150% of the poverty line, the association between Black race and APOT remained significant (11.75-minute APOT increase for the 3.3% increase in Black residents; 95% CI, 1.87 to 21.62; *P* = .02). This model, admittedly, carries over adjustment risk from underrecognized collinearity, colliders, and partial mediation. Figure 2 shows the estimated APOT for the range of Black resident proportions in the fully adjusted model, with covariates at their observed distributions. Figures 2 and 3 visualize the association between APOT and Black race, adjusted for confounders at their observed distributions. Figure 3 showed the interaction between Black race and 150% poverty by estimating adjusted APOT over the observed range of Black residents at both the 75th and 25th percentiles of 150% poverty among observed LEMSAs. A locally estimated scatterplot smoothing tool was used for visualization.

## Discussion

This study is the first we know of to reveal significant associations between APOT and community socioeconomic, demographic, and LEMSA characteristics in any population. Among the 5.9 million offloads in California between January 1, 2021, and June 30, 2023, we identified 11 characteristics that were significantly linearly associated with APOT, including Black race, single-parent households, and age. Applying regularized machine learning to better understand factors associated with APOT, we identified the proportion of residents of Black race, those aged 65 years and older, and ambulance offload volumes as significant variables associated with APOT. However, after hypothesis testing to evaluate suspected confounding, interaction, and mediation among the measures, the proportion of Black residents emerged as the major independent factor associated with longer APOT.

Notably, a California community with 1.6% Black residents (the 25th percentile among LEMSAs in CA) is estimated to have a 17.4-minute shorter APOT than a community with 4.9% Black residents (the 75th percentile among LEMSAs in CA). As longer APOT is associated with delayed access to ED care and reduced EMS availability to respond to other emergencies in the community, a better understanding of the interplay between race, socioeconomic status, and APOT is a public health priority. Black race remained independently associated with a substantially longer APOT after adjustment for confounding by age, socioeconomic characteristics, and LEMSA characteristics. The association between Black race and APOT was greatest at higher levels of poverty, indicating that each factor must be understood in the context of the other. The association between Black race and APOT, however, was significant independent of poverty and was not mediated by poverty. Other socioeconomic and household characteristics that were initially associated with longer APOT, such as the proportion of single-parent households, were no longer significantly associated after adjustment for Black race.

Our results also revealed a positive association between offload volumes and APOT, independent of Black race or economic disadvantage. This relationship may be attributable to an inadequate number of hospitals or ED staff to handle EMS systems with higher rates of 911 ambulance utilization or fewer available hospitals, specialty centers, or other transport destinations. The finding that APOT, on average, was higher in LEMSAs with a greater number of offloads may seem at odds with our finding that APOT was lower in LEMSAs with more people aged 65 years or older. An older population may be expected to use emergency services more frequently, leading to a greater number of offloads. We considered that those aged 65 years or older are eligible for Medicare and Social Security benefits, which may reduce the uninsurance rate and alleviate the economic strains that co-occur with longer APOT. The association between the proportion of residents aged 65 years or older and APOT, however, remained unchanged after adjustment for uninsurance and poverty, contradicting our hypothesis. Moreover, the association between APOT and those aged 65 years or older did not vary with different proportions of uninsurance or poverty and neither uninsurance nor poverty served as intermediates along the pathway linking age this age group and APOT. We did, however, find that being aged 65 years or older was no longer predictive of APOT after adjustment for both economic disadvantage and Black race, and that both economic disadvantage and Black race, alone and together, predicted decreased proportions of residents aged 65 years or older and increased proportions of those aged 17 years or younger. Similarly, the inverse association between being aged 65 years or older and total offloads was no longer significant after adjustment for Black or White race. In sum, our analyses suggest that LEMSAs with higher proportions of minors were more economically disadvantaged, had higher proportions of Black residents, and had longer APOT while LEMSAs with higher proportions of residents aged 65 years or older were less economically disadvantaged, had higher proportions of White residents, and shorter APOT.

Our main finding, that communities with higher proportions of Black residents are associated with longer APOT, highlights a potential mechanism behind delays in access to emergency care for Black communities. Although the association between delays in care and worse outcomes has been most clearly shown for time-sensitive conditions such as traumatic injury,^17^ stroke,^18^ and ST-segment elevated myocardial infarction,^19^ longer APOT has been broadly associated with increased length of stay and increased mortality.^7,20^ After trauma, for example, every 10-minute increase in prehospital time is associated with a 4% increase in the adjusted odds of hospital mortality.^15^ Taken together with extensive research showing that Black individuals have among the worst health outcomes of racial and ethnic groups in the US^21,22^ and are more likely to activate 911 emergency ambulance services,^23^ the results of this study suggest a role for APOT along the chain that links race to disparate outcomes.

The mechanism through which communities with a higher proportion of Black residents, especially economically disadvantaged communities, experience longer APOT warrants further study. Recognizing that race is a social construct, we consider institutional factors like understaffed, underfunded EDs or too few ED or hospital beds in hospitals that primarily serve Black patients.^24,25^ Whether due to inaccessibility or mistrust, inadequate outpatient preventative care or delays in seeking care^26^ may lead Black patients to rely more heavily on emergency services^23^ and present with greater medical complexity for comparable health utilization rates,^27^ plausibly contributing to racial disparities in APOT. Implicit bias and discrimination may entwine with each of these contributors.

### Limitations

This study has several important limitations. First, APOT interpretation is limited by the wide variability in EMS tracking systems and patient care report software used to collect these data. This variability may bias our findings in ways that, absent more granular EMS-level data, are hard to estimate. The California Emergency Medical Services Information System and National Emergency Medical Services Information System are important repositories of EMS data, but reporting must be standardized to ensure their quality and comparability in future studies. Next, our analyses are aggregated over time and by LEMSA. Temporal trends such as seasonality or the impact of the COVID-19 pandemic are unknown. As COVID-19 has been shown to exacerbate health disparities in other contexts,^28,29^ it might have similarly widened the disparity we find here. We previously showed that LEMSAs with the longest mean APOTs also had greater monthly APOT swings,^2^ suggesting that the LEMSAs with the longest APOTs either experience more capacity strain or are more vulnerable to it. Time-varying data may elucidate the dynamic associations between race, socioeconomic status, offload patterns, and APOT to examine whether APOT’s susceptibility to health strain is disparate. Additionally, the factors associated with APOT may differ by rurality. Greater housing units, mobile homes, households per capita, and more residents aged 65 years or older may be associated with rural settings while greater total offloads, LEMSA population size, Black and minoritized race proportions, proportion of minors, economic disadvantage, and crowded living conditions may describe urbanity. Unmeasured confounders related to urbanity may be contributing to our results. For example, urban trauma centers receive more offloads per ED bed, are more likely to be understaffed, have longer APOT, and may be more likely to serve economically disadvantaged and racially minoritized communities. Unfortunately, as many LEMSAs are made up of both urban and rural communities, incorporating rurality or urbanity measures was not practical in this study. Relatedly, our data were not granular enough to determine how EMS or hospital-level factors, such as offloads per ED bed, staffing, or urbanity may affect APOT.

## Conclusions

Our study reveals several demographic, socioeconomic, and LEMSA characteristics significantly associated with APOT in California LEMSAs. The proportion of Black residents was found to have the strongest association with longer APOT, and while the association between Black race and APOT could not be understood without measures of socioeconomic disadvantage, it also could not be fully explained by them. Given that APOT is an important measure of a community’s access to definitive emergency care with implications for hospital length of stay and mortality, our findings suggest that APOT may be an important measure of disparities to target. Further research on more granular hospital and EMS-level data and on mechanisms of this racial disparity is warranted.

## Notes

Supplement 1. **eMethods. **Additional details on methods for data collection **eTable 1.** Table of adjustment variables **eTable 2. **Crude associations between APOT (minutes) and the demographic, socioeconomic, household/housing, and LEMSA characteristics with which it was statistically significant **eTable 3. **Select regression models estimating associations with APOT
eReferences.

Supplement 2.
Data Sharing Statement
