# Supplementary material for: Racial and Socioeconomic Disparities in California Ambulance Patient Offload Times
Source: JAMA Netw Open. 2025 May 15;8(5):e2510325. doi: 10.1001/jamanetworkopen.2025.10325 (PMC12082368; doi:10.1001/jamanetworkopen.2025.10325)
Supplement: Supplement 1. — eMethods. Additional details on methods for data collection eTable 1. Table of adjustment variables eTable 2. Crude associations between APOT (minutes) and the demographic, socioeconomic, household/housing, and LEMSA characteristics with which it was statistically significant eTable 3. Select regression models estimating associations with APOT eReferences. [file jamanetwopen-e2510325-s001.pdf]

Supplemental Online Content

Shteyler VM, Feldmeier M, Bagay RJG, Ballard D, Colwell C, Hsia RY. Racial and socioeconomic disparities in ambulance patient offload times. *JAMA Netw Open*. 2025;8(5):e2510325. doi:10.1001/jamanetworkopen.2025.10325

eMethods. Additional details on methods for data collection

eTable 1. Table of adjustment variables

eTable 2. Crude associations between APOT (minutes) and the demographic, socioeconomic, household/housing, and LEMSA characteristics with which it was statistically significant

eTable 3. Select regression models estimating associations with APOT

eReferences.

This supplemental material has been provided by the authors to give readers additional information about their work.

© 2025 Shteyler VM et al. *JAMA Network Open*.

eMethods. Additional details on methods for data collection

We sought to collect hospital-level data on ambulance patient offload times (APOT) from local emergency medical services (EMS) agencies for January through March 2023 as this was not available through EMSA during this period. Data collection occurred from February 1, 2024, to April 17, 2024. The steps were as follows:

1. From February to March 2024, we sought to elicit initial information on APOT reporting practices by local EMS agency. We conducted internet searches of all public-facing local EMS agency websites and searched for mentions of APOT data reporting using key words such as "ambulance patient offload times," "APOT," "ambulance offload delay," and "EMS reports." We also reviewed previous APOT data reports and press releases. We found that the availability and presentation of public-facing APOT data varied significantly by local EMS agency— from no mention of APOT to weekly reports and interactive APOT dashboards. We collected contact information for 2-3 individuals in each local EMS agency who could provide APOT data, namely EMS administrators, medical directors, data analysts, and statisticians. If email contacts were unavailable, we called the general local EMS agency contact to request further information.
    a. From local EMS agency websites, we downloaded any publicly available datasets that included monthly hospital-level 90th percentile APOT times and offload volumes for January to March 2023 (5 of 34 local EMS agencies).
2. Next, we contacted all 34 local EMS agencies to request hospital-level data for January through March 2023. Through email correspondences, we gathered this data for 28/34 local EMS agencies.
3. Given that datasets were sent in various document formats (PDFs, CSVs, Excel sheets) as well as time-formatting for APOT times, we proceeded to standardize all local EMS agency data collected into an Excel format that could be merged with the EMSA data.

© 2025 Shteyler VM et al. *JAMA Network Open*.

eTable 1. Table of adjustment variables

**LEMSA characteristics**	Total offloads<br>Mean annual offloads per 1000 people<br>LEMSA area (square miles)<br>LEMSA population
**Socioeconomic Status**	Below 150% poverty line<br>Unemployed<br>Burdened by housing cost<br>No High School diploma<br>No health insurance
**Household Characteristics**	Age 65 and older<br>Age 17 and younger<br>Civilian with a disability<br>Single-parent household<br>Limited English language proficiency<br>No internet
**Racial and Ethnic Categories**	Hispanic or Latino<br>Black or African American (not Hispanic or Latino)<br>Asian (not Hispanic or Latino)<br>American Indian or Alaska Native (not Hispanic or Latino)<br>Native Hawaiian or Pacific Islander (not Hispanic or Latino)<br>Two or more races<br>Additional races (not Hispanic or Latino)<br>Minoritized race or ethnicity
**Housing-type and Transportation**	Housing units per capita<br>Households per capita<br>Multi-unit structures per capita (10 or more units)<br>Mobile homes per capita<br>Crowding (More people than rooms)<br>No vehicle<br>Group quarters

Adjustment variables related to socioeconomic status, household characteristics, race/ethnicity, and housing type/transportation were obtained from the CDC Social Vulnerability Index.[1,2]

© 2025 Shteyler VM et al. *JAMA Network Open*.

eTable 2. Crude associations between APOT (minutes) and the demographic, socioeconomic, household/housing, and LEMSA characteristics with which it was statistically significant

**LEMSA population characteristic**	**APOT (95% CI)**	
		**p-value**
**LEMSA population***	0.42 (0.12–0.72)	0.002
**Offloads***	0.42 (0.20–0.64)	<0.001
**Black or African American race**	3.37 (1.85–4.88)	<0.001
**Minoritized race or ethnicity**	0.50 (0.22–0.78)	0.001
**Single-parent households**	18.07 (5.66–30.48)	0.008
**Age 65 or older**	-2.33 (-3.46– -1.20)	<0.001
**Age 17 or younger**	1.79 (0.35– 3.23)	0.020
**Crowding**	7.81 (0.65–14.99)	0.040
**Households per capita**	-1.71 (-3.13– -0.28)	0.026
**Housing units per capita**	-1.10 (-1.92– -0.27)	0.014
**Mobile homes per capita**	-4.69 (-8.40– -1.92)	0.019

*APOT change for every 100,000 people or 10,000 offloads. Otherwise, APOT change reflects a 0.01 increase in population fraction

© 2025 Shteyler VM et al. *JAMA Network Open*.

eTable 3. Select regression models estimating associations with APOT

Model	Coefficient (95% CI)	p-value
**LASSO penalization (with unbiased CI)**		
log Black race	6.7 (0.37–12.1)	0.041
log offloads	5.2 (1.9–8.5)	0.007
log age ≥ 65	-24.0 (-39.0– -7.8)	0.009
**Black race—Economic disadvantage interaction**		
log Black race	66.50 (28.3—104.7)	0.002
log 150% poverty	118.41 (40.9—195.9)	0.005
Interaction log Black race and log 150% poverty	29.05 (7.7—50.3)	0.012
**Economic disadvantage mediating Black race—APOT**		
Mediated effect	0.75 (0.58—2.9)	0.33
Direct effect	14.8 (8.6—21.0)	<0.001
Total effect	15.5 (9.5—21.6)	<0.001
Proportion mediated	0.05 (-0.05—0.19)	0.33
**Single-parent households adjusted for Black race**		
log Black race	13.8 (8.4—19.2)	<0.001
Single-parent households	901.0 (-103.7—1905.8)	0.09
**Age ≥ 65 adjusted for Black race—Economic disadvantage interaction**		
log age ≥ 65	-12.2 (-32.5—8.1)	0.25
log 150% poverty	98.6 (14.9—182.3)	0.03
log Black race	56.8 (15.6—98.0)	0.01
Interaction log Black race and log 150% poverty	24.5 (2.1—47.0)	0.04

© 2025 Shteyler VM et al. *JAMA Network Open*.

eReferences

1. CDC SVI Documentation 2022 | Place and Health | ATSDR. May 22, 2024. Accessed October 8, 2024. https://www.atsdr.cdc.gov/placeandhealth/svi/documentation/SVI_documentation_2022.html

2. Dillingham S, Jarmin R, Fontenot AE, Stempowski DM. Understanding and Using American Community Survey Data: What All Data Users Need to Know.

© 2025 Shteyler VM et al. *JAMA Network Open*.
