# Supplementary material for: Racial and Socioeconomic Disparities in California Ambulance Patient Offload Times
Source: JAMA Netw Open. 2025 May 15;8(5):e2510325. doi: 10.1001/jamanetworkopen.2025.10325 (PMC12082368; doi:10.1001/jamanetworkopen.2025.10325)
Supplement: Supplement 2. — Data Sharing Statement [file jamanetwopen-e2510325-s002.pdf]

Data Sharing Statement

Shteyler. Racial and Socioeconomic Disparities in California Ambulance Patient Offload Times. *JAMA Netw Open*. Published May 15, 2025. doi:10.1001/jamanetworkopen.2025.10325

Data
Data available: No
